# Connecting knowledge and action: recommendations to leverage Guatemala's scientific diaspora to address water management challenges

**DOI:** 10.3389/frma.2026.1843700

**Published:** 2026-07-16

**Authors:** Marco E. Franco, Susana García, Giovanna Gatica-Domínguez, Ninoshka López-Xalín, Virginia Mosquera, Claudia S. Romero-Oliva, Katherine Santizo, Axel García y García

**Affiliations:** 1 Department of Environmental Toxicology, Swiss Federal Institute of Aquatic Science and Technology, Eawag, Dübendorf, Switzerland; 2 Instituto de Investigación en Ciencias Naturales y Tecnología (IARNA), Universidad Rafael Landívar, Guatemala, Guatemala; 3 Department of Business and IT, University of South-Eastern Norway, Kongsberg, Bø Telemark, Norway; 4 Organization of Women in Science for the Developing World (OWSD), Guatemala National Chapter, Guatemala; 5 International Nutrition Consultant, Lisbon, Portugal; 6 MarineGEO Program, Smithsonian Environmental Research Center, Edgewater, MD, United States; 7 Centro de Estudios Atitlán (CEA), Universidad del Valle de Guatemala, Sololá, Guatemala; 8 Department of Forest Ecology and Management, Swedish University of Agricultural Sciences, Umeå, Sweden; 9 Observatorio Económico Sostenible (OES), Universidad del Valle de Guatemala, Ciudad de Guatemala, Guatemala; 10 European Chemical Industry Council (Cefic), Brussels, Belgium; 11 Department of Agronomy and Plant Genetics, University of Minnesota, St. Paul, MN, United States

**Keywords:** freshwater, governance, human health, pollution, sanitation, scientific diasporas

## Abstract

Guatemala possesses substantial freshwater availability compared to many Latin American countries, yet it faces a profound water crisis marked by unequal access, widespread pollution, and weak governance. A large segment of the population—particularly rural indigenous communities—still lacks safely managed water and sanitation. This paradox stems from a lack of an overarching water law, resulting in decades of chronic under-investment in water infrastructure and institutional fragmentation. Water quality is further compromised by unregulated land-use change, agricultural runoff, industrial discharge, inadequate solid waste management, and untreated wastewater, all of which pose significant risks to human wellbeing, freshwater biodiversity, and ecosystem resilience. These interconnected challenges are exacerbated by a significant gap between scientific knowledge and policymaking, which has hindered effective, evidence-based solutions. In this context, the Guatemalan scientific community, including its global diaspora, represents an untapped resource to bridge these gaps. Through structured integration and collaboration, diaspora scientists can collectively contribute their expertise and analytical tools to complement and strengthen national water management capacities. Therefore, drawing from the scientific diaspora's interdisciplinary experience across water resources, agriculture, environmental sciences, and public health, this perspective outlines opportunities and recommendations to connect diaspora scientists with local scientists and practitioners, civil communities, government institutions and policymakers, and the industrial sector. By fostering inclusive, science-policy interfaces to co-develop locally grounded solutions and platforms for capacity building aligned with sustainable development goals, Guatemala can move toward more equitable, sustainable, and evidence-driven water management.

## 1 Introduction

Guatemala is a lower-middle-income country in Central America characterized by relatively abundant freshwater resources, including extensive river networks, lakes, groundwater reserves, and high annual rainfall in many regions (Carrera and Mosquera, 2023; Villatoro, 2023). In principle, this hydrological wealth, even when unevenly distributed across space and seasons, should provide a strong foundation for water security and sustainable development. In practice, however, Guatemala faces profound and persistent challenges in ensuring equitable, reliable, and safe access to water services for its population (Kenny, 2025). According to a recent report from Human Rights Watch, approximately 40% of Guatemalans—particularly indigenous communities living in rural areas and in conditions of extreme poverty—lack access to running water in their homes, and continue to experience chronic water insecurity, poor water quality, and inadequate sanitation services (Human Rights Watch, 2025). This reality reflects a systematic failure to convert water availability into human wellbeing.

These inequities stem from historical, structural, and institutional limitations, including under-investment in water infrastructure, fragmented governance at all levels, and weak regulatory frameworks that have collectively undermined effective water management (Cheatham et al., 2022). Limited technical capacity and insufficient integration of scientific and technical knowledge into decision-making processes further constrain policy implementation. As a result, water-related challenges intersect with broader issues such as poverty, public health, food security, and environmental degradation, disproportionately affecting vulnerable populations.

Guatemala's water challenges also reflect global concerns outlined in the United Nations Sustainable Development Goals (SDGs). In particular, SDG 6, which targets the availability and sustainable management of water and sanitation services, and SDG 10, which focuses on reducing inequalities within and among countries. Despite international commitments and several attempts to establish a national water law guiding nation-wide efforts toward improving water management (Cheatham et al., 2022), advancement continues to lag. These persistent gaps underscore the need for context-specific, inclusive, and science-informed approaches that account for Guatemala's social and institutional realities (Human Rights Watch, 2025). Addressing these structural limitations requires not only political will or institutional reforms, but also the strategic mobilization of expertise through participatory governance processes capable of bridging knowledge gaps and supporting evidence-based action. These dimensions are mutually reinforcing: the effective mobilization of expertise depends on institutional openness and governance structures that enable collaboration between scientists, communities, and decision-makers.

In this perspective, we highlight that the Guatemalan scientific diaspora constitutes an underutilized but strategically positioned stakeholder in efforts to address national water management challenges. The emigration of highly qualified Guatemalans, in pursuit of advanced education and professional opportunities, has fostered expertise across many disciplines, including hydrology, engineering, public health, environmental sciences, and the social sciences. Beyond this technical expertise, the Guatemalan scientific diaspora offers a distinct comparative advantage through its comprehensive knowledge with the country's sociocultural, linguistic, and institutional context while simultaneously maintaining access to international research networks, technologies, and collaborations. This dual positioning facilitates the translation of knowledge into locally relevant and culturally informed approaches to water governance. Examples of these advantages include the participatory science approach to evaluate the factors associated with the occurrence of metals and “forever chemicals” in Guatemala City's tap water, led by Guatemalan and U.S.-based institutions (Hoponick-Redmon et al., 2022) and a study about surface water-groundwater interactions led by a Guatemalan diaspora scientist at a Portuguese university in collaboration with a Guatemalan private sector institution (Paíz et al., 2023).

Indeed, previous work by (Bonilla et al. 2022b) highlighted the value of networking, engagement, and shared learning within the Guatemalan scientific diaspora. The present perspective advances this discussion by proposing a structured mobilization framework focused specifically on water-related challenges. Rather than emphasizing diaspora connectivity alone, we outline actionable pathways for integrating scientific expertise into science-policy interfaces, governance processes, capacity building, and long-term institutional collaboration. Our framework builds upon broader scholarship on knowledge mobilization and emphasizes the importance of sustained and locally grounded implementation processes.

When effectively connected with local scientists and communities, the industrial sector, and policymakers, members of the scientific diaspora can help bridge science-policy gaps by supporting evidence-based decision-making and strengthening national capacity for water governance. The proposed conceptual and actionable framework would mobilize scientific expertise through collaborative, transparent, and accountable dialogue. Importantly, the implementation of this mobilization framework would require sustained institutional support, public-sector coordination, and dedicated resource allocation mechanisms. By explicitly linking scientific knowledge and technical practices to policy development, the proposed guidance aims to move Guatemala toward durable, equitable, and sustainable water management solutions.

## 2 Pressing challenges in water resource management in Guatemala

Despite national access to improved water supply increased from 87% in 2000 to 91% in 2014 (Pérez et al., 2024; World Bank, 2018), these statistics mask significant disparities in service quality, reliability, and coverage, particularly in rural areas. Nationwide, only about 15% of drinking water delivered through networked systems undergoes disinfection to meet minimum standards (Lentini, 2010). Even in urban areas such as Guatemala City, where supply is comparatively better, drinking water systems depend heavily on groundwater, and recent studies report overextraction of key aquifers and reduced recharge due to urbanization and pollution from untreated wastewater (Saravia-Matus et al., 2023).

Wastewater treatment coverage also remains limited. By 2021, only 11% of municipalities had wastewater treatment plants (WWTPs; (SEGEPLAN 2023)), despite regulatory obligations requiring municipal wastewater treatment (i.e., Government agreement 236-2006). Many water, sanitation, and hygiene (WASH) systems operate without adequate planning, monitoring, or trained personnel, contributing to operational failures and environmental pollution (Caballero et al., 2024).

Sanitation disparities are also pronounced, with coverage estimated at 81% in urban areas but only 26% in rural regions (World Bank, 2018). Limited access to safe WASH services increases exposure to waterborne pathogens, diarrheal diseases, and parasitic infections, particularly among children (Braghetta, 2006; Kuper et al., 2018). Studies in rural areas have reported coliform and *Escherichia coli* contamination in nearly 80% of household drinking water sources (Estrada et al., 2016).

Water management challenges also affect agriculture, a cornerstone of Guatemala's economy. Inefficient water use, limited irrigation infrastructure, and increasing competition for water resources threaten agricultural productivity (Kondash et al., 2021; Pinillos et al., 2025). These pressures are intensified by climate change, projected to reduce national water yield by at least 13% under future scenarios (Pinillos et al., 2025).

Pollution from agricultural runoff, mining activities, urban waste, and emerging pollutants further degrades water quality. More than 90% of surface waters show evidence of pollution (Carrera and Mosquera, 2023; Gil-Rodas et al., 2021; Mosquera et al., 2026), contributing to ecosystem degradation, harmful algal blooms such as those observed in Lake Atitlán (Neher et al., 2021), and significant plastic pollution exported primarily through the Motagua River—Guatemala's longest and most polluted river (Meijer et al., 2021).

## 3 Mobilizing Guatemalan scientists and practitioners for water solutions

Guatemala reflects global patterns where scientific knowledge remains fragmented across sectors and institutions, resulting in limited integration into water governance and decision-making processes. Its scientific and technological capacity remains comparatively limited within Latin America, reflecting long-standing underinvestment in education and restricted institutional support for scientific careers. Available international indicators show that Guatemala has one of the lowest densities of researchers in the region, with UNESCO data reporting fewer than 100 researchers per million inhabitants, a number substantially below regional averages (UNESCO, 2025). However, migration patterns associated with limited research opportunities and postgraduate training have contributed to the growth of a highly skilled scientific diaspora across geographic regions. Existing initiatives such as *Converciencia*, coordinated by the National Secretariat of Science and Technology (SENACYT), have demonstrated the potential of diaspora engagement by facilitating scientific exchange and collaboration between local actors and researchers abroad since 2005 (Bonilla et al., 2022a). More importantly, much of the diaspora's expertise intersects at the water-human-environment interface, making Guatemala's water crisis particularly well-suited to demonstrate how integrated approaches can benefit and strengthen decision-making around human health, ecosystem integrity, and sustainable livelihoods.

Mobilizing this collective capacity requires deliberate mechanisms for collaborative dialogue grounded in integrative principles recognizing the interdependence of human, animal, plant, and environmental systems (Lebov et al., 2017). Accordingly, the mobilization of scientific diaspora expertise should encourage interdisciplinary collaboration across environmental sciences, engineering, hydrology, public health, epidemiology, agriculture, and social sciences to support integrated and preventive approaches to water governance. This perspective is particularly relevant in regions facing similar contexts as in Guatemala, where water contamination, ecosystem degradation, food insecurity, and public health vulnerabilities frequently intersect within highly unequal social and environmental contexts.

**Figure 1:**
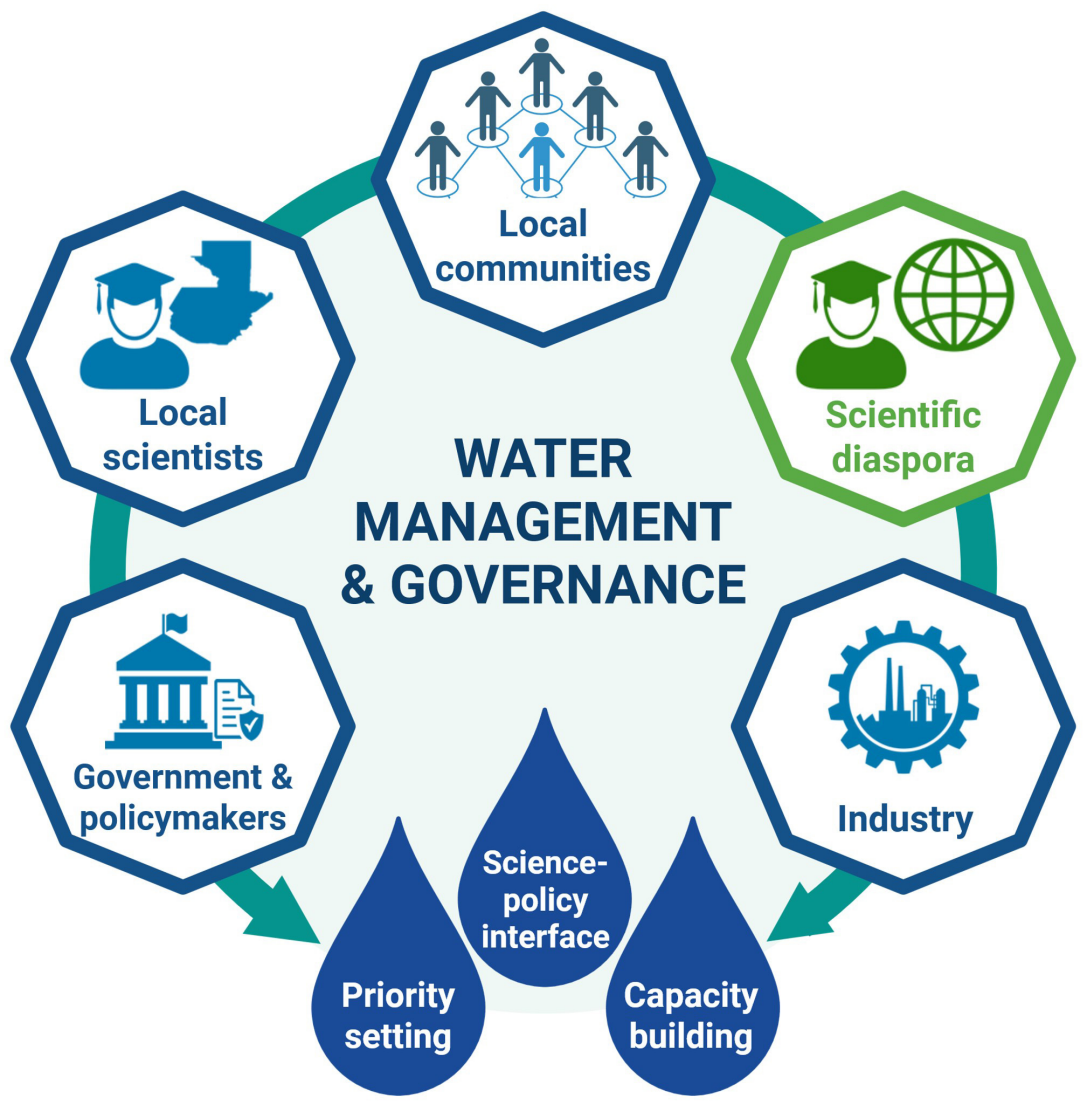
Visualization of the integrated cross-sector expertise and knowledge to collectively design and execute the three core pillars of the proposed collaborative framework toward more efficient water management and governance in Guatemala.

Open, transparent, and inclusive platforms can bring together scientists in Guatemala and the diaspora, alongside community leaders, indigenous authorities, the industrial sector, and policymakers, to collaboratively identify priorities, build capacity, and co-design water solutions that integrate wellbeing, environmental, economic, and social dimensions through science-policy interfaces (Figure 1). Such platforms can facilitate cross-sectoral learning, align research agendas with national and local needs, and ensure that water management strategies address co-benefits and trade-offs across human health, food systems, and ecosystems, provided that participation is accompanied by clearly defined roles, decision-making responsibilities, and accountability mechanisms. Literature on knowledge co-creation and participatory science emphasizes that effective interfaces between science, policy, and society depend on legitimacy, mutual accountability, shared ownership of knowledge production, and sustained institutional support (Norström et al., 2020; Wyborn et al., 2019). In an institutionally fragmented context such as Guatemala, establishing such platforms requires clearly defined governance arrangements and mechanisms that promote equitable participation among communities, scientists, and policymakers.

Similar efforts to mobilize scientific expertise and strengthen science-policy collaboration have been documented in multiple regions of the world, particularly within low- and middle-income countries facing complex environmental governance challenges. For example, scientific diaspora engagement initiatives in Latin America and the Caribbean countries, South Africa, and India have demonstrated the potential of transnational scientific networks to support national development priorities through policy advising, research collaboration, capacity building, and technology transfer (Echeverría-King et al., 2022; Tejada and Bolay, 2010). Likewise, participatory water governance and knowledge co-production initiatives, in regions such as North America, have highlighted the importance of long-term trust-building, local ownership, institutional coordination, and sustained funding to maintain effective science-policy collaboration (Armitage et al., 2011).

Importantly, the scientific diaspora plays a supportive role rather than an executive one. Their influence is in strengthening locally led processes through technical advising, knowledge translation, international networking, comparative policy learning, and capacity building. The effectiveness of these contributions depends on sustained collaboration to integrate scientific evidence into governance and regulatory processes.

Central to this effort is meaningful engagement with communities—particularly indigenous and rural populations whose livelihoods, wellbeing and cultural identities are closely linked to Guatemala's water resources. Local knowledge systems, traditional water governance practices, and lived experiences offer critical insights into environmental change, disease risks, and resource management that complement scientific and technical approaches. Collaborative frameworks should therefore promote voluntary participation, cultural respect, and shared decision-making to avoid perceptions of externally imposed solutions.

Furthermore, particular attention should also be given to gender equity and intersectional inclusion within participatory governance processes. Women in indigenous communities and rural populations often experience disproportionate impacts from access to inadequate water and sanitation services, and environmental degradation, while simultaneously facing barriers to participation in scientific, technical, and policy decision-making arenas (Harris, 2009). Likewise, women scientists and underrepresented groups within diaspora networks may encounter structural limitations affecting access to leadership opportunities, funding, and international collaboration. Integrating gender-responsive and socially inclusive approaches may therefore strengthen both the legitimacy and effectiveness of collaborative water governance initiatives, as was discussed in the context of water politics in Bolivia (Laurie, 2011).

Altogether, the Guatemalan scientific diaspora can play a catalytic role by contributing interdisciplinary expertise, comparative insights from other geographic regions, and access to international research networks while operating under locally defined priorities and institutional leadership. Collaborative research initiatives, pilot projects, and capacity-building programs allow scientists within Guatemala and abroad to bridge persistent gaps between research, governance, policy, and implementation. Prioritizing long-term, reciprocal partnerships will be essential for translating scientific knowledge into durable and locally grounded water solutions.

## 4 Recommendations for the scientific diaspora to contribute its expertise to water-related national needs, lawmaking, and regulation

**Figure 2:**
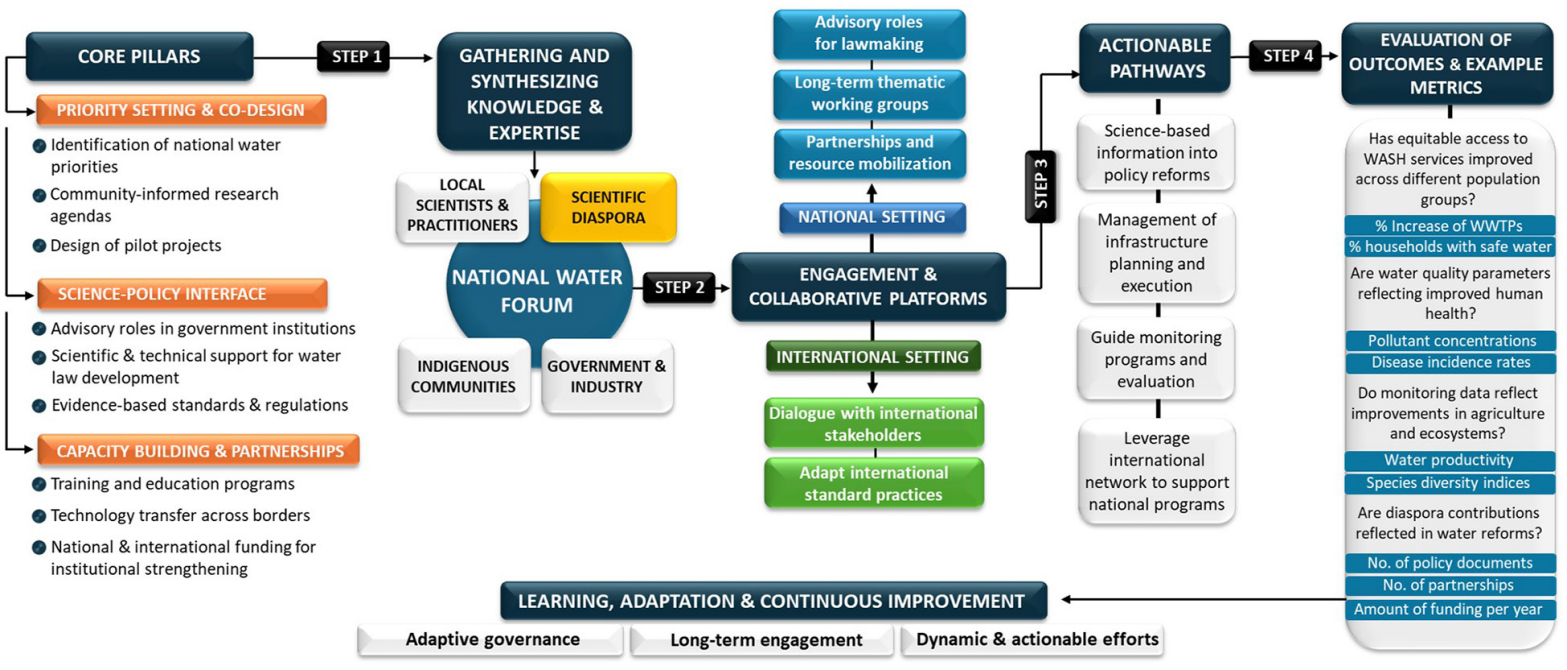
Graphical representation of the recommended framework for mobilizing the Guatemalan scientific diaspora to support national water management through priority setting and co-design, science-policy interfaces, and capacity building. The flow chart illustrates a stepwise framework to translate scientific expertise into actionable outcomes. The process begins with the establishment of a national forum that convenes local and diaspora scientists, indigenous leaders, government institutions, industry, and practitioners to identify shared priorities and co-design research and policy agendas. Outcomes from this forum feed into collaborative national platforms, where diaspora scientists engage in advisory roles and thematic working groups that support infrastructure planning, water quality monitoring, and data collecting related to public health, agriculture, and socio-economic indicators. These collaborations are reinforced through international partnerships and academic networks, enabling technology transfer, training programs, and resource mobilization. The framework then advances through actionable pathways, including science-informed policy reforms, technical guidance for infrastructure development, integrated monitoring programs that link water quality, ecosystem health, and public health indicators, and strategic interactions with international networks to support national needs. Finally, the framework incorporates an evaluation and feedback stage that assesses impacts on equitable access to WASH services, including the percentages of active WWTPs and households with safe water in both urban and rural areas, improvements in water quality measured by concentrations of pollutants and water-related disease incidence, agricultural productivity and ecosystem (biodiversity) resilience, and metrics to assess whether diaspora contributions are reflected in water reforms. Continuous monitoring and participatory evaluation create feedback loops that foster learning, adaptation, and long-term engagement, enabling adaptive and inclusive water governance in Guatemala.

The central contribution of this perspective is to propose a framework outlining actionable recommendations and guidance about how the Guatemalan scientific diaspora can meaningfully support national water management efforts. This framework is organized around three complementary core pillars that are strategically aligned to ensure a logical and tangible path forward to transition knowledge to action (Figure 2). These core pillars entail: (i) the collective identification of national, regional (i.e., Latin America and the Caribbean) and global priorities, and the co-design of agendas across sectors, (ii) the establishment of science-policy interfaces to introduce co-advisory roles within local government organizations to provide technical support and to foster scientific know-how, and (iii) the establishment of long-term capacity building initiatives and partnerships through resource mobilization.

Although this framework emphasizes the mobilization of scientific expertise, its implementation fundamentally depends on the active participation of governmental institutions responsible for water governance, public investment, and regulation. Government bodies would play a central role in convening stakeholders, supporting institutional continuity, facilitating access to public data, and allocating the financial and administrative resources necessary to sustain collaborative platforms, technical working groups, and monitoring systems. Resource mobilization may include public funding mechanisms, international cooperation programs, university partnerships, and co-financing agreements with civil society and industry.

Guatemala has experienced localized and sector-specific initiatives that illustrate both the potential and the limitations of collaborative water governance approaches. Examples include participatory watershed governance efforts in the Naranjo River Basin and the Tacaná Watersheds, where municipalities, local organizations, and external technical partners collaborated to strengthen integrated water resource management and local governance (Barchiesi and Córdoba, 2016; Gaviño-Novillo et al., 2020). Community-based water governance in municipalities like Chiantla and Huehuetenango has demonstrated the importance of local participation and traditional management systems while also revealing barriers related to institutional fragmentation, weak legal frameworks, and limited resources (Gamboa et al., 2007). Initiatives including the USAID-supported I-WASH program and governance-oriented WASH projects by organizations such as Helvetas have emphasized multi-level coordination, local capacity building, and resource mobilization as critical elements for improving water governance in Guatemala (Kenny, 2025). However, initiatives designed to institutionalize the long-term mobilization of scientific diaspora expertise into national water governance processes remain rare. Our proposed framework aims to advance more structured, sustained, and science-informed collaboration mechanisms adapted to Guatemala's institutional realities.

### 4.1 Priority setting and co-design

As the first step, we propose the creation of a national cross-sector forum to collectively identify water-related priorities at the national, regional and global levels. This forum should bring together local and diaspora scientists alongside indigenous leaders and advocates, government agencies, municipalities, industry, and civil society organizations. By integrating diverse and transnational expertise, the forum would support the co-design of research, policy, and regulatory agendas aligned with the country's identified priorities.

The proposed forum should adopt a transparent, inclusive, and evidence-based process, including structured dialogues, thematic working groups, and consensus-building processes capable of translating scientific knowledge into actionable guidance for policymakers. Existing outreach platforms, such as the International Symposium of Continental Waters in the Americas—a professional gathering in Guatemala that began as a space to share scientific research in Lake Atitlán in 2014 and evolved to an international meeting—could serve as a platform to identify pilot projects that integrate scientific innovation with community-based solutions. Over time, the forum could function as a permanent mechanism for revising priorities, informing regulatory processes, and sustaining long-term engagement with scientific diaspora in strengthening national water management.

### 4.2 Science-policy interfaces and co-advisory roles

The actionable outcomes of the national water forum must be operationalized through structured science-policy interfaces. In this context, scientists from the Guatemalan diaspora can assume advisory roles aligned with their expertise and with priorities identified by local stakeholders. Their participation should complement—and not duplicate or replace—the work of scientists and practitioners based in Guatemala. Diaspora scientists should work within permanent, official groups. Rather than directly changing water policies, they can help provide high quality data that policymakers need. Furthermore, to support effective integration, thematic working groups can be established within local organizations to provide targeted technical support on issues such as infrastructure improvement, research and monitoring programs, and equitable access to safe water.

These science-policy interfaces require clear procedures for decision support and co-advisory mechanisms that ensure continuous communication between scientific experts and governmental and non-governmental institutions. Sustaining long-term cooperation requires official coordination rather than e.g., *ad hoc* interactions. Examples include (i) regular multi-stakeholder forums, (ii) dedicated liaison officers, (iii) official agreements between universities and government, and (iv) technical committees with clear duties and reporting structures. Sharing data regularly and communicating publicly ensures continuity despite political turnover and limited resources.

Formalized co-advisory roles leveraging the international network of diaspora scientists would also enable evidence-based guidance for water-related planning, regulatory development, and institutional strengthening. Open channels of dialogue with international stakeholders would reinforce the proposed interfaces by facilitating the adoption and adaptation of global standards, technical best practices, and lessons learned from countries that have successfully advanced integrated water management. Such structures can reduce duplication of efforts, prevent “reinventing the wheel” actions, and support the translation of scientific recommendations into regulatory and operational practice. Experiences from participatory governance and co-production initiatives have demonstrated that sustaining these interfaces often requires dedicated intermediaries, institutional leaders, facilitation processes, and long-term relationship building among stakeholders (Cash et al., 2003; Reed et al., 2018). As such, the development of effective science-policy interfaces should be viewed as an adaptive governance process rather than a fixed institutional structure.

### 4.3 Long-term capacity building and partnership through resource mobilization

Advancing water governance also requires sustained capacity-building initiatives supported by strategic partnerships and adequate resource mobilization. Integrating the scientific diaspora into these efforts creates opportunities for collaboration with international organizations that can expand access to funding, technology transfer, and specialized training. Partnerships between government agencies, universities, research institutions, industries, indigenous communities, and civil society organizations are essential to move long-term initiatives forward.

Academic institutions play a central role in this process by supporting training programs, facilitating joint research, and offering technical tools that strengthen national expertise. Diaspora scientists can serve as bridges between institutions, enabling joint supervision of students and early-career researchers as well as co-developed curricula and research platforms. These activities help ensure that newly acquired knowledge remains embedded in national institutions.

Capacity building also includes support for robust monitoring systems that inform adaptive management. Both locally based and diaspora scientists can contribute to the design of monitoring frameworks that integrate water quality, ecosystem health, and public-health indicators. By promoting data transparency, open-access tools, and institutional learning, these long-term partnerships enhance Guatemala's ability to implement evidence-based policies, strengthen WASH systems, and sustain progress well beyond the initial interventions.

## 5 Evaluation of outcomes and impact: learning, adaptation, and continuous improvement

Evaluating the effectiveness of the actionable pathways within the proposed framework requires a structured, evidence-based approach that emphasizes the continuous improvement and adaptation of monitoring, evaluation, accountability, and learning activities (i.e., MEAL approach). Core evaluation metrics derived from systematic data collection initiatives (e.g., national census) should assess whether access to safe and reliable WASH services have become more equitable across geographic, ethnic, and socioeconomic groups, and quantify the magnitude and distribution of these changes over time. Through integrative approaches, evaluation must incorporate indicators linking environmental quality, ecosystem conditions, and human wellbeing.

For example, longitudinal monitoring of water quality parameters—including microbial, chemical, and emerging pollutants—can provide critical evidence of environmental and public health gains through trend analyses, including tracking disease incidence in children in conjunction with locally grounded health services. Quantitative indicators may include (i) the percent increase of operating WWTPs, (ii) the concentrations of fecal coliforms and nutrient pollution levels in water bodies, (iii) the proportion of households with reliable access to safe water and sanitation services across different population groups, and (iv) incidence rates to estimate the number of emerging cases of disease occurring during a defined period.

In parallel, these integrated monitoring programs should also examine whether water-related interventions enhance agricultural productivity and the resilience of the ecosystems and biodiversity assemblages. Key metrics could include, respectively, the yearly water productivity and Shannon-Wiener diversity indices in areas with moderate to high risk of pollution, including measurements of species richness and abundance of sensitive biota (e.g., aquatic macroinvertebrates). Together, these measures can reveal whether scientific inputs are translating into tangible improvements at the human-environment interface.

Beyond biophysical and service delivery outcomes, evaluation must also examine institutional, governance, and capacity-building impacts. Key questions include whether science-informed policy reforms and regulatory updates have reduced fragmentation in water governance, improved enforcement, and contributed to measurable reductions in inequality. Furthermore, the effectiveness of diaspora engagement itself should be assessed by tracking indicators such as the uptake of scientific advice in policy decisions, given by e.g., the number of policy documents and technical guidelines with contributions from diaspora scientists, the number of diaspora members affiliated with Guatemalan institutions (e.g., universities), and the amount of external or co-financing secured for collaborative networks as proxies for the sustainability of partnerships. The strengthening of local technical capacity may include the number of jointly delivered training activities, co-authored scientific publications, student mentorship initiatives, and improvements in institutional monitoring and analytical capabilities. The continuity of monitoring and data-sharing platforms could be assessed through indicators such as the operational persistence of monitoring stations, the frequency of data updates, the number of participating institutions, and the maintenance of accessible long-term environmental and health datasets.

Importantly, evaluation should be iterative and participatory, involving local communities, policymakers, and scientists in interpreting results and adjusting strategies accordingly. Lastly, by embedding feedback loops into the proposed system, all participants can ensure continuous learning, adaptation, and improvement, thus guaranteeing adaptive governance, long-term engagement, and the refinement of dynamic and actionable frameworks that transform evaluation from a retrospective exercise into a driver of adaptive, inclusive, and resilient water management.

## 6 Discussion and closing remarks

Guatemala's water challenges exemplify the complex and deep interconnections between environmental degradation, public health risks, food insecurity, and social inequities (Kondash et al., 2021; Kuper et al., 2018; Trudeau et al., 2018; Villatoro, 2023). Despite relatively abundant freshwater resources, fragmented governance, aging and insufficient infrastructure, widespread pollution, and unequal access continue to undermine water security, disproportionately affecting rural and indigenous populations. These challenges are further exacerbated by climate change and weak regulatory frameworks, underscoring that Guatemala's water crisis is not merely technical but fundamentally systemic. Addressing it requires coordinated governance, monitoring, and implementation across all sectors involved, and approaches that simultaneously consider human health, ecosystem integrity, and sustainable livelihoods (Pérez et al., 2024; Villatoro, 2023).

These processes are inherently political and institutional in nature, requiring negotiation among stakeholders, sustained public and private investment, regulatory enforcement, and mechanisms that foster trust between communities, scientists, and government institutions (Cash et al., 2003). Accordingly, the mobilization of diaspora expertise should be understood not as external to governance reform, but as part of broader efforts to strengthen participatory and evidence-informed institutional processes. Equity considerations should also remain central to the implementation of collaborative governance frameworks. International scholarship on water governance and sustainable development consistently demonstrates that socially inclusive and gender-responsive participation processes contribute to more equitable, legitimate, and durable environmental decision-making outcomes, particularly in contexts characterized by socio-environmental inequality and institutional exclusion (Arora-Jonsson, 2011).

We are confident that mobilizing the Guatemalan scientific diaspora, in close partnership with locally based scientists, communities, and policymakers, offers a concrete pathway to bridge long-standing gaps between knowledge and action for water management and governance in the country. Studies of knowledge co-production and participatory governance have shown that science-informed policy processes are most effective when they incorporate locally grounded expertise, institutional legitimacy, sustained stakeholder engagement, and adaptive learning mechanisms (Wyborn et al., 2019). Nevertheless, the transformative potential of diaspora engagement should not be overstated. Sole scientific expertise cannot overcome structural barriers such as political instability, weak institutional coordination, and insufficient public investment and enforcement of regulations. Instead, diaspora engagement should be viewed as one enabling component within broader processes of institutional strengthening, participatory governance, and long-term policy reform. Mitigating existing barriers would require formalized advisory mechanisms, long-term institutional partnerships between diaspora networks and national organizations, dedicated funding mechanisms, multilingual and culturally sensitive engagement strategies, and participatory governance processes that strengthen trust and local ownership. These considerations have been highlighted as effective tools in similar efforts related to environmental management (Reed et al., 2018) and reinforce the importance of viewing diaspora mobilization as a gradual and adaptive governance process rather than a short-term institutional solution.

Our recommendations translate this vision into practice by outlining mechanisms for inclusive priority setting, strengthened science-policy interfaces, and sustained capacity building. The proposed actionable pathways demonstrate how diaspora expertise can be operationalized to support national water management efforts while reinforcing local ownership and institutional capacity, as shown in efforts involving scientific diasporas in other regions of the world (Echeverría-King et al., 2022; Tejada and Bolay, 2010).

Equally important, we emphasize evaluation, learning, and adaptation as central components of effective governance. Therefore, the feedback loops that assess equity, environmental quality, public health outcomes, and institutional performance can move Guatemala toward adaptive, evidence-based water management. Ultimately, leveraging the scientific and professional expertise of the diaspora could transform water from a source of vulnerability into a foundation for resilience, equity, and sustainable development, offering lessons that extend beyond Guatemala to other resource-rich yet institutionally constrained settings.

## Data Availability

The original contributions presented in the study are included in the article/supplementary material, further inquiries can be directed to the corresponding author/s.
